# A single center experience with publicly funded clinical exome sequencing for neurodevelopmental disorders or multiple congenital anomalies

**DOI:** 10.1038/s41598-021-98646-w

**Published:** 2021-09-27

**Authors:** Ben Pode-Shakked, Ortal Barel, Amihood Singer, Miriam Regev, Hana Poran, Aviva Eliyahu, Yael Finezilber, Meirav Segev, Michal Berkenstadt, Hagith Yonath, Haike Reznik-Wolf, Yael Gazit, Odelia Chorin, Gali Heimer, Lidia V. Gabis, Michal Tzadok, Andreea Nissenkorn, Omer Bar-Yosef, Efrat Zohar-Dayan, Bruria Ben-Zeev, Nofar Mor, Nitzan Kol, Omri Nayshool, Noam Shimshoviz, Ifat Bar-Joseph, Dina Marek-Yagel, Elisheva Javasky, Reviva Einy, Moran Gal, Julia Grinshpun-Cohen, Mordechai Shohat, Dan Dominissini, Annick Raas-Rothschild, Gideon Rechavi, Elon Pras, Lior Greenbaum

**Affiliations:** 1 The Danek Gertner Institute of Human Genetics, Sheba Medical Center, Tel Hashomer, Israel; 2 The Institute for Rare Diseases, Edmond and Lily Safra Children’s Hospital, Sheba Medical Center, 5265601 Tel Hashomer, Ramat Gan, Israel; 3 Talpiot Medical Leadership Program, Sheba Medical Center, Tel Hashomer, Israel; 4 Sackler Faculty of Medicine, Tel Aviv University, Tel Aviv, Israel; 5 The Genomics Unit, Sheba Cancer Research Center, Sheba Medical Center, Tel Hashomer, Israel; 6 The Wohl Institute for Translational Medicine, and the Sheba Cancer Research Center, Sheba Medical Center, Tel Hashomer, Israel; 7 Community Genetics, Public Health Services, Ministry of Health, Jerusalem, Israel; 8 Internal Medicine A, Sheba Medical Center, Tel Hashomer, Israel; 9 Pediatric Neurology Unit, Edmond and Lily Safra Children’s Hospital, Sheba Medical Center, Tel Hashomer, Israel; 10 Pediatric Neurology Unit, Edith Wolfson Medical Center, Holon, Israel; 11 The Joseph Sagol Neuroscience Center, Sheba Medical Center, Tel Hashomer, Israel

**Keywords:** Medical genetics, Molecular medicine, Neurodevelopmental disorders

## Abstract

Exome sequencing (ES) is an important diagnostic tool for individuals with neurodevelopmental disorders (NDD) and/or multiple congenital anomalies (MCA). However, the cost of ES limits the test's accessibility for many patients. We evaluated the yield of publicly funded clinical ES, performed at a tertiary center in Israel, over a 3-year period (2018–2020). Probands presented with (1) moderate-to-profound global developmental delay (GDD)/intellectual disability (ID); or (2) mild GDD/ID with epilepsy or congenital anomaly; and/or (3) MCA. Subjects with normal chromosomal microarray analysis who met inclusion criteria were included, totaling 280 consecutive cases. Trio ES (proband and parents) was the default option. In 252 cases (90.0%), indication of NDD was noted. Most probands were males (62.9%), and their mean age at ES submission was 9.3 years (range 1 month to 51 years). Molecular diagnosis was reached in 109 probands (38.9%), mainly due to de novo variants (91/109, 83.5%). Disease-causing variants were identified in 92 genes, 15 of which were implicated in more than a single case. Male sex, families with multiple-affected members and premature birth were significantly associated with lower ES yield (*p* < 0.05). Other factors, including MCA and coexistence of epilepsy, autism spectrum disorder, microcephaly or abnormal brain magnetic resonance imaging findings, were not associated with the yield. To conclude, our findings support the utility of clinical ES in a real-world setting, as part of a publicly funded genetic workup for individuals with GDD/ID and/or MCA.

## Introduction

Neurodevelopmental disorders (NDD), including global developmental delay (GDD) and intellectual disability (ID), constitute a wide and heterogeneous group of conditions affecting brain function, many of which are caused by a monogenic etiology^1,2^. While the diagnostic process for patients with NDD is often lengthy, the growing use of exome sequencing (ES) in the clinical setting has revolutionized the field of medical genetics^3–5^. Another group of patients who stand to benefit from ES are those with multiple congenital anomalies (MCA), especially when they remain undiagnosed following chromosomal microarray analysis (CMA)^6–8^. For these indications, not only may ES raise novel diagnostic options or assist in confirming a suspected clinical diagnosis in a relatively short time period, it could also spare unnecessary tests and medical procedures, and occasionally has the potential to change the patient’s management^9,10^.

ES yield in undiagnosed individuals affected by NDD (with or without additional features) and/or MCA varies between different cohorts and centers, and is estimated at around 25–35%, depending on patient selection criteria^7–9,11,12^. Several other factors probably influence the yield, including ES type (singleton vs. trio), study design and recruitment procedures, population characteristics, quality of available clinical data and the availability of previous genetic workup^1,8^.

From a perspective of a large tertiary referral center in Israel, we describe herein our real-world experience with a publicly funded program pursuing clinical ES for 280 consecutive undiagnosed individuals with GDD/ID and/or MCA. We assessed the potential impact of demographic and clinical factors on ES yield, and discuss insights gained from the first three years of experience utilizing this diagnostic workflow.

## Methods

### Patient selection

In general, ES is not part of the publicly funded health services in Israel, and is usually privately paid for by patients and their families. In 2018, the Israeli Ministry of Health (MOH) launched a program to fund trio clinical ES for families in which the undiagnosed proband met one or more of the following criteria:

1. Moderate to profound GDD with impairment in at least two developmental domains/ID (defined by Developmental Quotient /Intelligence Quotient [DQ/IQ] score < 55);
2. Mild GDD/ID (DQ/IQ score < 70) with either epilepsy or a major congenital anomaly.
3. Two (or more) major congenital anomalies in different body systems (defined as MCA), and clinical suspicion of a monogenic disorder.

Prior to ES, probands were referred for Fragile X and CMA testing, which are publicly funded for these indications. If clinically significant findings were not detected, the patients were considered eligible for ES.

Cognitive evaluation was performed using standardized assessment tools selected according to each participant's age and developmental stage, such as the Mullen scale for early learning^13^, the Wechsler Preschool and Primary Scale of Intelligence (WPPSI)^14^, the Wechsler Intelligence Scale for Children (WISC)^15^, and others. The DQ or IQ scores were specifically documented in the genetic counseling reports for each patient. MCA included congenital heart defects, brain malformations, anomalies of limbs, eyes, kidneys and urinary tract, digestive system, genitalia and more.

This retrospective analysis included all families that received genetic consultation at the Danek Gertner Institute of Human Genetics or the Institute for Rare Diseases at the Sheba Medical Center (SMC), between January 2018 and December 2020, if they had no previous molecular diagnosis, met the inclusion criteria detailed above and eventually underwent publicly funded ES. Notably, hundreds of pediatric and adult patients are consulted by our service annually, and only a minor part of them are eligible for MOH funding of trio ES. Patients who do not meet these criteria may choose to pay privately for ES.

### Recruitment process

Initial genetic counseling was conducted by a physician who is board certified in medical genetics. Evaluation included obtaining detailed family history and medical information, as well as a physical examination of the proband. As abovementioned, prior to ES, Fragile X and CMA were pursued. In cases with a high level of suspicion of a specific syndrome, testing of known founder mutations or targeted sequencing of one or two relevant genes were required first (e.g. for tuberous sclerosis, Rett syndrome, *PTEN* etc.). In a few other cases, additional relevant tests were undertaken (e.g., imprinting or trinucleotide repeats tests). Only patients who met the inclusion criteria and were not molecularly diagnosed by these tests were included in the ES program. Their medical information, including detailed clinical summary and Human Phenotype Ontology (HPO) terms, were submitted for approval to the Department of Community Genetics at the Israeli MOH.

Once approved, the clinical ES costs were covered by the MOH and participation was offered to families at no charge. Probands and their parents arrived at the genetic institute, and blood was obtained for DNA extraction, following an explanation about the risks and benefits of ES and provision of written informed consent. For patients aged < 18 years or patients who were unable to provide an informed consent, this was obtained from their parents or legal custodians.

Trio (proband and both parents) ES was pursued as the default option. When one parent was unavailable, ES included a healthy sibling instead, or was performed as a duo format (proband and single parent). In a limited number of cases, ES was carried out as a quartet (two affected siblings and both parents). In these cases, the extra sequencing charges were covered by SMC.

ES was provided as a clinical service, and turn-around time to final test report was up to 2–3 months from the date of submission. Finally, all families were given a concluding genetic counseling session, that included a thorough explanation about the molecular diagnosis (if found), further possible genetic tests and/or a recommendation for revision of the ES data in 12 months (for unsolved cases), and potential treatment options in select cases. Families were informed about recurrence risk and options of future family planning, including prenatal or pre-implantation genetic testing.

The current study was performed in accordance with relevant guidelines and regulations, and was approved by the Institutional Review Board (IRB) of the Sheba Medical Center.

### ES procedure and analysis

Since the sequencing itself was not performed at SMC, DNA samples were sent to one of three approved service laboratories. One of the following three capture kits were used for ES: Twist Human Core Exome Kit (Twist Bioscience, San Francisco, CA, USA); SureSelect Human All Exon Kit V6 (Agilent, Santa Clara, CA, USA); or xGen Exome Research Panel V2 Kit (Integrated DNA Technologies, Coralville, Iowa, USA). In all cases, sequencing runs were performed on a HiSeq2500 sequencing machine (Illumina, San Diego, CA, USA).

Bioinformatics analysis was carried out at the Genomics Unit of the Sheba Cancer Research Center. Reads were aligned to the UCSC human reference assembly (hg19) with BWA-MEM algorithm (BWA v.0.7.15)^16^. Variants were called by applying GATK variant caller version 3.8 using guidelines recommended by the Broad Institute^17^, and annotated with KGGSEQ^18^. Further annotation and filtration steps were taken using various public databases, the Human Gene Mutation Database (HGMD) and internal databases by application of in-house custom scripts. For 3 families, ES was analyzed in another laboratory (outside SMC).

Variants were interpreted according to the American College of Medical Genetics and Genomics (ACMG) guidelines^19^, and were classified as pathogenic, likely-pathogenic, variants of uncertain significance (VUS), likely benign or benign.

### Molecular diagnosis

In SMC, the Genomics unit staff and the medical geneticists collaborate closely to maximize the integration of laboratory and clinical expertise. Therefore, the final decision about molecular diagnosis was approved by both the Genomics unit team and the referring geneticist, based on the variants frequency and classification (ACMG recommendation), expected inheritance pattern and correlation between the suggested disease-causing gene and the clinical phenotype. The SMC in-house exome dataset (of more than 5000 exomes at the end of 2020) has appropriate representation of the Israeli population, including Ashkenazi and non-Ashkenazi Jewish ancestries. This is important for correctly assessing variant frequencies in populations not well represented in external databases. In some families, segregation analysis with additional members was required to reach a more definitive diagnosis.

De novo variants were defined as variants detected in the proband sample and not in parental samples (pathogenic strong 2 criteria, according to ACMG). When only a single parent was included in the analysis, we were unable to classify the variant as such. De novo variants were validated by Sanger sequencing if coverage of wild-type allele in the parents was insufficient.

In patients with multiple or inconclusive findings, the final diagnosis was only reached after consulting with additional experts. For the 3 families whose ES was performed outside SMC, the final decision about diagnosis was made by the referring geneticist, based on similar consideration.

### Statistical analysis

To calculate the diagnostic rate, each family was analyzed and counted as a single case.

We evaluated the association of several demographic and clinical factors with overall diagnostic yield and rate of de novo variants. Demographic factors included: proband sex, age at ES submission, age of each parent at proband birth, parental consanguinity and existence of multiple-affected family members (first degree with similar phenotype). Clinical factors included NDD (GDD/ID), MCA, and coexistence of epilepsy, autism spectrum disorder (ASD), microcephaly (head circumference < 3 centile), abnormal brain Magnetic Resonance Imaging (MRI) findings, if done, and history of prematurity (defined as birth before completion of 37 gestation weeks). ASD was diagnosed by a neurologist or a psychiatrist, according to the Diagnostic and Statistical Manual of Mental Disorders (DSM–5) criteria, using tools appropriate to each individual condition (such as the Social Communication Questionnaire (SCQ)^20^, Social Responsiveness Scales (SRS)^21^ and the Autism Diagnostic Observation Schedule (ADOS)^22^).

Pearson chi-square, Fischer’s exact or Mann–Whitney tests were used for statistical analysis, as appropriate. A *p* < 0.05 (two-sided) was considered statistically significant. However, since type I error may occur due to multiple testing, the findings of these analyses should be interpreted with caution.

## Results

The final sample included 280 consecutive cases. Their demographic and clinical characteristics are shown in Table 1. GDD/ID was diagnosed in 252 probands (90.0%), 64 had MCA (22.9%) and 176 were males (62.9%). Mean age at ES submission was 9.3 ± 10.1 years, ranging from 1 month to 51 years. In 44 of cases (15.7%), the proband was older than 18 years. Mean maternal and paternal age at birth was 31.6 ± 5.4 and 34.3 ± 6.1 years, respectively. In 11 families (3.9%), consanguinity was noted. Two hundred and fifty-two families (90.0%) performed trio ES, while in 22 (7.9%) and 6 (2.1%) a quartet exome and duo exome were pursued, respectively.

**Table 1﻿:** Demographic and clinical characteristics of 280 probands with GDD/ID and/or MCA for which exome sequencing was pursued, and comparison between probands with and without a subsequent molecular diagnosis.

	Total (%)	Proband with molecular diagnosis	Proband without molecular diagnosis	*p* value
Number of cases (%)	280	109 (38.9)	171 (61.1)	
Age of proband, mean (SD) years	9.3 (10.1)	10.2 (10.6)	8.8 (9.7)	0.298
Adult proband (> 18 years), N (%)	44 (15.7)	20 (18.3)	24 (14.0)	0.334
Sex, N (%)				
Male	176 (62.9)	59 (54.1)	117 (68.4)	**0.016**
Female	104 (37.1)	50 (45.9)	54 (31.6)	
Paternal age at birth, mean (SD) years	34.3 (6.1)	35.1 (6.5)	33.8 (5.9)	0.115
Maternal age at birth, mean (SD) years	31.6 (5.4)	32.2 (5.3)	31.3 (5.4)	0.216
Jewish origin, N (%)	276 (98.6)	106 (97.2)	170 (99.4)	0.303
Consanguinity, N (%)	11 (3.9)	4 (3.7)	7 (4.1)	1.00
ES format, N (%)				0.395
Trio	252 (90.0)	101 (92.7)	151 (88.3)	
Quartet	22 (7.9)	7 (6.4)	15 (8.8)	
Duo	6 (2.1)	1 (0.9)	5 (2.9)	
Cases with VUS, N (%)	74 (26.4)	16 (14.7)	58 (33.9)	**< 0.001**
Multiple-affected family (first degree), N (%)	54 (19.3)	10 (9.2)	44 (25.7)	**0.001**
GDD/ID, N (%)	252 (90.0)	99 (90.8)	153 (89.5)	0.713
MCA, N (%)	64 (22.9)	21 (19.3)	43 (25.1)	0.253
Epilepsy, N (%)	128 (45.7)	55 (50.5)	73 (42.7)	0.203
ASD, N (%)	75 (26.8)	23 (21.1)	52 (30.4)	0.086
Microcephaly, N (%)	77 (27.5)	26 (23.9)	51 (29.8)	0.275
Abnormal brain MRI findings, N (%)	84 (30.0)	30 (27.5)	54 (31.6)	0.470
Premature birth, N (%)	43 (15.4)	10 (9.2)	33 (19.3)	**0.022**

Statistically significant values (*p* < 0.05) are given in bold.

ASD, autism spectrum disorder; ES, exome sequencing; GDD, global developmental delay; ID, intellectual disability; MCA, multiple congenital anomalies; MRI, magnetic resonance imaging; N, number; NDD, neurodevelopmental delay; SD, standard deviation; VUS, variant of uncertain significance.

Molecular diagnosis was found in 109 of the 280 probands (38.9%), 91 with a de novo disease-causing variant (83.5% of the overall diagnosis). In 77 probands, an autosomal de novo heterozygous variant was observed, and in 14 a heterozygous/hemizygous variant in X chromosome. Recessive inheritance was seen in 9 cases (8.3%) (homozygous or compound heterozygous variants), inherited autosomal dominant in 5 (4.6%) and in 3 (2.8%) we observed variants compatible with X-linked inheritance from the mother. In one case a duo exome was performed, and the pattern of inheritance could not be conclusively determined.

Notably, in probands with GDD/ID, the diagnostic rate was 39.3% (99/252), and 86 of the resolved cases had de novo variants (86.7%).

In total, disease causing variants were found in 92 genes. Of these, 15 genes were observed in more than one family, due to different variants. These included *AHDC1, ARID1B, FOXP1, KANSL1, KCNC2, KCNQ2, KMT5B, MAP2K1, MED13L, OPHN1, PPP2R5D, SMC1A, STXBP1, TCF4* and *TCF20*. In two genes (*ARID1B* and *SMC1A*), pathogenic variants were found in three unrelated probands. Of the 111 pathogenic/likely-pathogenic variants, 55 (49.5%) were missense, 23 (20.7%) frameshift, 19 (17.1%) stop gain, 9 (8.1%) splice site variants and 5 (4.5%) of other types (i.e. in-frame deletions, deletion of an entire exon, etc.). 94 variants (84.7%) were located on autosomal chromosomes and 17 (15.3%) on X chromosome. A comprehensive list of all identified pathogenic or likely pathogenic variants, including demographic, clinical and molecular characteristics, is presented in Supplemental Table 1. Variants classified as VUS were observed in 74 (26.4%) probands.

In order to evaluate the association of demographic and clinical variables with the diagnostic yield (Table 1), we compared diagnosed to undiagnosed probands. In cases with a molecular diagnosis (109), the rate of male proband was significantly lower (*p* = 0.016) compared to 171 cases who remained undiagnosed. The number of families with multiple-affected members (first degree) or premature birth of the proband was significantly lower in the group of resolved compared to the unresolved cases (*p* = 0.001 and *p* = 0.022, respectively). Other demographic and clinical factors- including MCA and coexistence of epilepsy, ASD, microcephaly or abnormal brain MRI- did not differ between the groups.

Finally, we compared the 91 probands with de novo variants to all other 189 (including 23 resolved cases with inherited variants and one with undetermined inheritance). The rate of male probands (*p* = 0.003), parental consanguinity (*p* = 0.018), families with multiple-affected members (*p* < 0.001) and premature birth (*p* = 0.034) were lower in the de novo group, compared to all of the other cases. Higher rates of epilepsy were found among the de novo variant group (*p* = 0.031). Other factors did not significantly differ between the groups. Interestingly, both maternal and paternal age at pregnancy were older in the de novo group, but this was not statistically significant (*p* = 0.076 and *p* = 0.061, respectively) (Table 2).

**Table 2:** Comparison between probands found to harbor a disease-causing de novo variant and all other probands (undiagnosed or diagnosed with non de novo variant).

	Proband with de novo variant	All other probands	*p* value
Number of cases (%)	91 (32.5)	189 (67.5)	
Age of proband, mean (SD) years	10.5 (10.7)	8.8 (9.8)	0.136
Adult proband (> 18 years), N (%)	16 (17.6)	28 (14.8)	0.551
Sex, N (%)			
Male	46 (50.5)	130 (68.8)	**0.003**
Female	45 (49.5)	59 (31.2)	
Paternal age at birth, mean (SD) years	35.3 (6.5)	33.8 (5.9)	0.076
Maternal age at birth, mean (SD) years	32.6 (5.3)	31.2 (5.3)	0.061
Jewish origin, N (%)	91 (100)	185 (97.9)	0.308
Consanguinity, N (%)	0	11 (5.8)	**0.018**
ES format, N (%)			0.067
Trio	87 (95.6)	165 (87.3)	
Quartet	4 (4.4)	18 (9.5)	
Duo	0	6 (3.2)	
Cases with VUS, N (%)	13 (14.3)	61 (32.3)	**0.001**
Multiple-affected family (first degree), N (%)	6 (6.6)	48 (25.4)	**< 0.001**
GDD / ID, N (%)	86 (94.5)	166 (87.8)	0.081
MCA, N (%)	16 (17.6)	48 (25.4)	0.145
Epilepsy, N (%)	50 (54.9)	78 (41.3)	**0.031**
ASD, N (%)	21 (23.1)	54 (28.6)	0.331
Microcephaly, N (%)	20 (22.0)	57 (30.2)	0.151
Abnormal brain MRI findings, N (%)	26 (28.6)	58 (30.7)	0.717
Premature birth, N (%)	8 (8.8)	35 (18.5)	**0.034**

Statistically significant values (*p* < 0.05) are given in bold.

ASD, autism spectrum disorder; ES, exome sequencing; GDD, global developmental delay; ID, intellectual disability; MCA, multiple congenital anomalies; MRI, magnetic resonance imaging; N, number; NDD, neurodevelopmental delay; SD, standard deviation; VUS, variant of uncertain significance.

## Discussion

We aimed to evaluate the yield of publicly funded clinical ES for patients with GDD/ID and/or MCA, in the real-world setting of a tertiary medical genetic counseling service in Israel. The overall diagnostic rate was 38.9% (109/280), mainly due to de novo variants (91/109, 83.5%).

The study sample is based on consecutive cases from 36 months of experience in a single referral center. The cohort size is relatively small compared to other ES studies for GDD or ID, which may include thousands of participants^23,24^. Nevertheless, the design of our study is unique in several ways, and offers interesting insights.

First, it is built on a publicly (MOH) funded clinical ES program, performed for medical diagnosis purposes as the primary goal, and not in the context of a research project. All families with consultations at our center during the 2018–2020 period that met the inclusion criteria were offered ES. We assume that due to financial considerations, a substantial number of these families would not have been able to pursue ES if required to cover the costs by themselves, out-of-pocket. As of 2020, trio ES pricing per customer in SMC is about 2000 USD. Indeed, a recent review of the effects of out-of-pocket and private pay in clinical genetic testing reaffirms this notion, and further suggests that such barriers might cause disparities in clinical outcomes for patients, based on their financial resources^25^.

Second, the inclusion criteria were stringent. The current study was based on careful phenotyping by skilled clinicians, completion of CMA for all cases, and additional genetic testing in a few specific cases, prior to ES. Singleton ES was not allowed, and trio ES was the default option, as the best and probably the most rapid strategy to detect de novo variants.

Recently, a meta-analysis by Srivastava et al. recommended ES as a first-tier test for individuals with NDD, with CMA as a next step^9^. However, the diagnostic workflow evaluated in this study, in agreement with the Israeli MOH guidelines, was not designed to assess ES as a first-tier test.

In hindsight, some of the syndromes detected could have been diagnosed clinically without necessitating ES, for example Neurofibromatosis type 1, Cardiofaciocutaneous syndrome, Osteogenesis imperfecta, and Ellis Van Creveld syndrome. However, even for these conditions, as well as in others, a differential diagnosis still exists with genetic heterogeneity, and the manifestations are not always entirely explained by the clinically suspected syndrome. Therefore, trio ES may be the preferred diagnostic approach even in these cases, to spare time and financial resources.

Finally, the study population was typical of the Jewish population seen in tertiary centers in Israel, and included mainly non-consanguineous families (96.1%). Nearly all ES samples were analyzed by a single bioinformatics laboratory (using a large in-house database which represents the local population), working in close collaboration with the clinical team and routinely discussing the results to improve diagnostic accuracy. The combination of all these factors probably contributed to the relatively high diagnostic rate in our sample.

A major limitation of this work is the relatively small sample size, as acknowledged. This is partially balanced by the strict patient inclusion criteria and the careful phenotyping. The diagnostic rate (38.9%) is comparable to previously reported studies of similar phenotypes^7,11,12^. Since sample collection is ongoing, future data from larger sample size will be available for analysis in the upcoming years, hopefully in collaboration with additional medical centers in Israel that provide publicly funded ES. This could also include a revision of ES data for unsolved cases or even whole-genome sequencing for these individuals. In addition, the rate of VUS (26.4%) in the current study was relatively high, but this may be reduced with future ES revisions. Another limitation is that sequencing was performed by three laboratory services using different capture kits, and not by a single facility (or in-house). Finally, we did not evaluate the impact of the molecular diagnosis on medical interventions for patients (for example, changes in drug treatment in cases of epilepsy).

In a more general context, we assume that despite these limitations, our findings constitute a good estimate of ES yield in individuals with similar indications of GDD/ID and/or MCA. As mentioned, funding is still a barrier to genetic testing for many families. Health service providers worldwide have different attitudes toward offering advanced genetic testing and its role in the diagnostic process. This study shows that when clinical ES is part of a publicly funded health service, is combined with transparent and clear inclusion criteria, is accessible to the population and accompanied by genetic counseling—it has a clear benefit.

Overall, the results emphasize the major role of de novo variants in the etiology of GDD/ID and MCA. De novo variants were found in 32.5% of probands and represent 83.5% of all cases with a molecular diagnosis. These findings are consistent with previous studies of their contribution to NDD^1,5,26,27^. Both the paternal and maternal age (at proband birth) may impact the detection of de novo variants, as we observed a trend towards older paternal (*p* = 0.076) and maternal (*p* = 0.061) age in families with de novo variants, compared to all other families (with or without a molecular diagnosis). For paternal age, this is consistent with the well-described evidence indicating that advanced paternal age might confer risk of NDD, due to increased occurrence of de novo variants^1,28–30^. Interestingly, recent studies have also raised evidence of an increased risk of de novo variants with increased maternal age, albeit to a lesser effect size when compared to paternal age^31,32^.

Detection of de novo variants has the potential to influence family planning of probands’ parents and sibling, and can provide reassurance of low recurrence risks in future pregnancies of the parents. Nevertheless, the possibility of gonadal mosaicism was conveyed to the parents, and for this reason we routinely recommended prenatal diagnosis in future pregnancies for the specific variants found.

Interestingly, 44 of the 280 probands (15.7%) in our sample were 18 years of age or older at ES submission. In this adult subgroup, a molecular diagnosis was reached in 23 probands (52.3%). This underscores the role of clinical ES in older patients with NDD/MCA, who frequently have undergone a previously negative diagnostic odyssey for several years. For this often-overlooked patient population, ES is a crucial addition to the clinician’s toolbox, as previously suggested^33^ and as reaffirmed in our study.

Examining possible associations of demographic and clinical characteristics with ES diagnostic yield revealed a number of intriguing results. However, part of them could be spurious, resulting from relatively small sample size and multiple testing. Premature birth was significantly associated with a lower diagnostic rate. This suggests that the phenotypic features (including NDD) might be explained, at least in part, by the preterm birth and its consequences, rather than a monogenic disorder. Diagnostic yield was also significantly lower in families with a male proband and multiple-affected individuals. The later finding may be attributable to the high rate of de novo (versus inherited) variants among the diagnosed cases, or perhaps to diagnoses being more easily achieved in multiple-affected families earlier in the genetic evaluation process, prior to ES introduction. Higher rate of epilepsy was seen in probands with de novo variants, potentially due to involvement of epileptic encephalopathy which is caused by this type of variants in many cases^27,34,35^.

The list of 92 genes that harbor disease-causing variants in our study included several genes for which evidence of their involvement in GDD/ID and/or epileptic encephalopathies have accumulated in recent years and are well-established, with numerous reported cases. Examples of such genes are *STXBP1* and *DDX3X*, the latter suggested to account for as many as 1–3% of ID in females^36^. In contrast, some of the other genes had only recently been identified as disease causing, with a limited number of described patients. For instance, *YWHAG* in which de novo heterozygous missense variants were implicated in NDD with early-onset seizures^37,38^. For this and other genes, our results further confirm an association with the disease, add to the current knowledge regarding the phenotype and expand the known mutational spectrum.

In a few of our patients, the detection of variants in genes not yet associated with disease in humans at the time of exome interpretation, contributed to the discovery of novel genetic syndromes that were published recently, following data sharing and matching with international collaborators. This was the case for proband 129 (Supplementary Table 1) that harbored a de novo heterozygous variant in *PPP2CA,* now implicated in syndromic intellectual disability^39^; and for proband 232, presenting with skeletal anomalies, found to be homozygous for a variant in *SCUBE3*^40^. These cases underscore an important aspect in which the ES approach may be superior to targeted gene panel sequencing, and may contribute to the identification of unrecognized, rare inherited disorders. Moreover, they highlight the importance of data sharing between clinicians and researchers worldwide, via matchmaking nodes^41^.

To conclude, our results support the important role of publicly funded clinical ES in the diagnosis of cases of GDD/ID and/or MCA in a real-world scenario. The majority of diagnosed cases are attributable to de novo variants. Further studies are required to identify the demographic and clinical factors which contribute to clinical ES yield.

## Supplementary Information

Supplementary Table 1.

## Data Availability

The datasets generated and analyzed during the current study are not publicly available due to patient privacy and confidentiality, but are available from the corresponding author on reasonable request.
