# Supplementary material for: A single center experience with publicly funded clinical exome sequencing for neurodevelopmental disorders or multiple congenital anomalies
Source: Sci Rep. 2021 Sep 27;11:19099. doi: 10.1038/s41598-021-98646-w (PMC8476634; doi:10.1038/s41598-021-98646-w)
Supplement: Supplementary file 1 — Supplementary Table 1. [file 41598_2021_98646_MOESM1_ESM.docx]

**Supplemental Table 1** - Clinical and molecular characteristics of individuals with GDD/ID and/or MCA for whom pathogenic or likely pathogenic variant(s) were detected by ES.

| **Proband serial number** | **Sex** | **Age at ES (years)** | **GDD/ID** | **Epilepsy** | **ASD** | **MCA** | **Additional phenotypic features** | **Molecular diagnosis (#MIM)** | **Gene** | **Chr** | **Variant** | **HGMD** | **ACMG criteria** | **Inheritance** | **Zygosity** |
| --- | --- | --- | --- | --- | --- | --- | --- | --- | --- | --- | --- | --- | --- | --- | --- |
| 3 | M | 23 | + | - | - | - | Dysmorphic facial features, scoliosis, strabismus, suspected hypoplastic CC | Xia-Gibbs syndrome (615829 ) | ***AHDC1*** | 1 | NM_001371928.1: c.2062C>T (p.R688*) | CM187519 | PVS1, PM2, PP5, PS2 | DN | Het |
| 98 | F | 8.5 | + | + | - | - | Macrocephaly, hearing impairment, thin CC | Xia-Gibbs syndrome (615829) | ***AHDC1*** | 1 | NM_001371928.1: c.1814_1819delCCAACGinsT (p.A605Vfs*36) | - | PVS1, PM2, PS2 | DN | Het |
| 165 | F | 4.5 | + | + | + | - | Microcephaly, coarse facial features, low posterior hairline, FTT, hypotonia, short second and 5th middle metacarpal, renal cystic disease | KBG syndrome (148050) | ***ANKRD11*** | 16 | ENST00000301030.10:  c.3309dup (p.D1104Rfs*2) | CI2014959 | PVS1, PM2, PP5, PS2 | DN | Het |
| 262 | F | 7 | + | + | - | - | Dysmorphic facial features, short stature, ADHD | Coffin-Siris syndrome 2 (135900) | ***ARID1A*** | 1 | NM_006015.6: c.3033G>T (p.L1011F) | - | PM2, PP5, PS2 | DN | Het |
| 29 | F | 3 | + | + | - | - | Dysmorphic facial features, FTT, partial agenesis of CC | Coffin-Siris syndrome 1 (135900) | ***ARID1B*** | 6 | NM_001374820.1: c.6377dup (p.S2126Rfs*46) | - | PVS1, PM2, PS2, PM4 | DN | Het |
| 146 | F | 1.5 | + | - | - | - | FTT, short stature, feeding difficulties, hypotonia | Coffin-Siris syndrome 1 (135900) | ***ARID1B*** | 6 | NM_001374820.1: c.2642_2645del (p.R881Tfs*46) | - | PVS1, PM2, PP5, PS2 | DN | Het |
| 159 | F | 5 | + | - | + | - | Coarse facial features, bushy eyebrows, hirsutism, partial hypoplasia of CC, persistent fetal fingertip pads | Coffin-Siris syndrome 1 (135900) | ***ARID1B*** | 6 | NM_001374820.1: c.3286_3287del (p.L1096Cfs*8) | - | PVS1, PM2, PS2 | DN | Het |
| 87 | M | 2.5 | + | - | + | - | Long philtrum, Greek-helmet shaped nose, left ear conductive hearing loss, broad thumbs | Mental retardation, autosomal dominant 26, (615834) | ***AUTS2*** | 7 | NM_015570.4: c.1611C>A (p.H537Q) | - | PM2, PM1, PS2 | DN | Het |
| 49 | F | 4.5 | + | - | - | - | Dysmorphic facial features, short stature, contractures | Intellectual developmental disorder with dysmorphic facies, speech delay, and T-cell abnormalities (618092) | ***BCL11B*** | 14 | NM_138576.4: c.2507G>A (p.S836N) | - | PM2, PS2 | DN | Het |
| 90 | M | 30 | + | - | - | - | Dysmorphic facial features, relative macrocephaly, ptosis, hypertelorism | Intellectual developmental disorder with dysmorphic facies and ptosis (617333) | ***BRPF1*** | 3 | NM_001319050.1: c.556C>T (p.Q186*) | CM199504^1^ | PVS1, PM2 | Inh | Het |
| 152 | M | 5m | + | + | - | - | Hypotonia, microstomia, bilateral adducted thumbs, bilateral single transverse palmar crease, bilateral syndactyly of II-III toes | Timoty syndrome (601005) | ***CACNA1C*** | 12 | NM_199460.3: c.4231G>T, p.V1411L | ^2^ | PM2, PM5, PP3, PS2 | DN | Het |
| 120 | F | 17.5 | + | + | - | - | Acquired microcephaly, hyperkinetic movement disorder, pontocerebellar hypoplasia | Mental retardation and microcephaly with pontine and cerebellar hypoplasia (300749) | ***CASK*** | X | NM_001126055.2: c.1623_1626del (p.T542Wfs*46) | - | PVS1, PM2, PP5, PS2 | DN | Het |
| 205 | M | 13 | + | + | + | + | Dysmorphic facial features, long face, hypotonia, strabismus, keratoconus, trigonocephaly, multiple congenital pigmented nevi, bilateral cryptorchidism, hypospadias, bilateral fifth finger clinodactyly | Congenital heart defects, dysmorphic facial features, and intellectual developmental disorder (617360) | ***CDK13*** | 7 | NM_003718.5: c.2201A>C (p.K734T) | - | PM2, PM5, PP3, PM1, PS2 | DN | Het |
| 46 | F | 6.5 | + | - | - | + | Microcephaly, atrial septal defect, suspected ocular albinism, mega cysterna magna, thin CC | Mental retardation, autosomal dominant 40 (616579) | ***CHAMP1*** | 13 | NM_032436.4: c.1995dup (p.S666*) | - | PVS1, PM2, PS2 | DN | Het |
| 42 | M | 18 | + | + | - | - | - | Raynaud-Claes syndrome (300114) | ***CLCN4*** | X | NM_001830.4: c.875G>A (p.W292*) | - | PVS1, PM2, PM4, PP4 | Inh | Hem |
| 170 | M | 1m | - | - | - | + | FTT, short limbs, multiple fractures (begining in utero), hydrocephalus | Osteogenesis imperfecta, type III (259420) | ***COL1A1*** | 17 | NM_000088.4: c.1273G>A (p.G425S) | CM930143 | PM2, PP3, PP2, PP5, PS2 | DN | Het |
| 247 | M | 6 | + | + | + | - | Apraxia, ADHD | Pitt-Hopkins like syndrome 1 (610042) | ***CNTNAP2*** | 7 | NM_014141.6: c.2151C>A (p.Y717*) | - | PVS1, PM2, | Inh | Hom |
| 133 | F | 2.5 | + | + | - | - | Widely spaced teeth, sleep disturbance | Neurodevelopmental disorder with spastic diplegia and visual defects (615075) | ***CTNNB1*** | 3 | ENST00000349496.11: c.646G>A (p.G216R) | - | PM2, PP2, PS2 | DN | Het |
| 97 | M | 10 | + | + | - | - | Smooth philtrum, small ears, laryngomalacia, flapping movements of hands and feet, happy demeanor, apraxia, suspected autonomic episodes | Mental retardation, X-linked, syndromic 15 (Cabezas type) (300354) | ***CUL4B*** | X | NM_00001330624.1: c.968_972del (p.I323Kfs*2) | CD070455 | PVS1, PM2, PP5 | DN | Hem |
| 80 | F | 2.5 | + | + | - | - | Incomplete lissencephaly, pachygyria | Lissencephaly, X-linked (300067) | ***DCX*** | X | NM_178152.3: c.788A>G (p.D263G) | CM131063 | PM2, PM1, PP2, PS2 | DN | Het |
| 102 | F | 17.5 | + | - | - | - | Long face, hypotelorism, astigmatism, acanthosis nigricans, ADHD | Intellectual developmental disorder, X-linked, syndrome, Snijders Blok type (300958) | ***DDX3X*** | X | ENST00000644876.2: c.1628C>T (p.S543L) | - | PM2, PP2, PS2 | DN | Het |
| 217 | M | 4 | + | - | - | - | High arched palate, widely spaced teeth, hypotonia, brachydactyly, joint hyperlaxity, self injurious behavior, sleep disturbance | Intellectual developmental disorder 62 (618793( | ***DLG4*** | 17 | ENST00000399506.9: c.1083G>A (p.S361=) | ^3^ | PM2, PP3, PS2 | DN | Het |
| 107 | F | 12.5 | + | + | - | - | Microcephaly, long face, wide mouth, small ears, white matter changes on brain MRI | Mental retardation, autosomal dominant 13 (614563) | ***DYNC1H1*** | 14 | NM_001376.5: c.9209C>T (p.P3070L) | - | PM2, PS2 | DN | Het |
| 257 | M | 8 | + | - | - | + | Microcephaly, dysmorphic facial features, hypertelorism, short stature, bilateral hearing impairment, dysgenesis of CC, hypospadias, clinodactyly, cubitus valgus | Mandibular dysostosis, Guion-Almeida type (610536) | ***EFTUD2*** | 17 | NM_004247.4: c.870-5_870-4del | - | PM2, PS2 | DN | Het |
| 150 | F | 5m | - | - | - | + | Agenesis of CC, colpocephaly of cerebral ventricles, bilateral optic nerve hypoplasia, dysplastic cerebellum, kinky hair, hypopigmentation (hair, eyes), anteverted nostrils, hemangioma, suspected contractures, elevated CK level | Vici syndrome (242840) | ***EPG5*** | 18 | NM_020964.3: c.1007A>G (p.Q336R) | CM163398, | PM2, PP5 | Inh | Hom |
| 142 | F | 2.5 | - | - | - | + | Vascular ring, renal vein thrombosis, aberrant right subclavian artery, liver cavernous hemangioma, portosystemic shunt, capillary malformation | Capillary malformation-arteriovenous malformation 2 (618196) | ***EPHB4*** | 7 | NM_004444.5: c.2605dup (p.Q869Pfs*77) | - | PVS1, PM2, PP4 | Inh | Het |
| 252 | M | 0.3 | - | - | - | + | Unbalanced AVSD, atrial septal defect, hypospadias, neonatal tooth, II-III toe syndactyly, polydactyly, nail dysplasia, short ribs | Ellis-van Creveld syndrome (225500) | ***EVC2*** | 4 | NM_147127.5: c.24del (p.R9Afs*52) | - | PVS1, PM2, PP4 | Inh | Hom |
| 59 | F | 3m | + | - | - | + | Hydrocephalus, meningomyelocele, facial dysmorphism, nasal aperture stenosis, external auditory canal atersia, partial agenesis of sacrum and additional skeletal findings | Antley-Bixler syndrome without genital anomalies or disordered steroidogenesis (207410) | ***FGFR2*** | 10 | ENST00000358487.10: c.1052C>G (p.S351C) | CM960650 | PM1, PP2, PM2, PP5 PS2 | DN | Het |
| 136 | M | 17 | + | + | - | - | Facial dysmorphism, tic disorder, polyhydramnios | Mental retardation with language impairment and with or without autistic features (613670) | ***FOXP1*** | 3 | NM_032682.6: c.1652+5G>A | - | PM2, PP5, PS2 | DN | Het |
| 164 | M | 3 | + | - | + | + | Posterior fossa Arachnoid cyst, ventriculomegaly, esotropia, strabismus, bilateral cryptorchidism, bilateral preauricular skin tags | Mental retardation with language impairment and with or without autistic features (613670) | ***FOXP1*** | 3 | NM_032682.6, c.1553G>A, (p.S518N) | - | PM2, PM1, PP3, PS2 | DN | Het |
| 184 | F | 37 | + | - | - | - | Speech apraxia and language disorder, palatal insufficiency | Speech-language disorder 1 (602081) | ***FOXP2*** | 7 | ENST00000350908.9: c.1690C>T (p.R564*) | CM170042 | PVS1, PM2 | Inh | Het |
| 93 | M | 2.5 | + | + | - | - | Downslanted palpebral fissures, short fingers, hyperactivity, behavioural problems | Developmental and epileptic encephalopathy 43 (617113) | ***GABRB3*** | 15 | NM_000814.6: c.675C>G, (p.F225L) | - | PM2, PM5, PP2, PS2 | DN | Het |
| 199 | M | 9.5 | + | + | - | - | Microcephaly, midfacial hypoplasia, macrostomia, stereotypic movements | Intellectual developmental disorder, autosomal dominant 6, with or without seizures (613970) | ***GRIN2B*** | 12 | NM_000834.5: c.1928T>C (p.L643P) | CM1813288 | PM2, PM1, PS2 | DN | Het |
| 22 | F | 8.5 | + | + | - | - | Microcephaly, dysmorphic facial features, FTT, hypotonia, cyclic vomiting | Cornelia de-Lange syndrome 5 (300882) | ***HDAC8*** | X | NM_018486.3: c.471T>G (p.D157E) |  | PM2, PP3, PP2, PS2 | DN | Het |
| 119 | F | 9.5 | + | + | - | + | Facial dysmorphism, hypodontia, macrocephaly, atrial septal defect, partial agenesis of CC, self injurious behaviour | Koolen-De Vries syndrome (610443) | ***KANSL1*** | 17 | NM_001193465.2: c.501_511dup (p.D171Vfs*35) |  | PVS1, PM2, PS2 | DN | Het |
| 192 | M | 16 | + | - | - | - | Hearing impairment, ADHD, short stature | Koolen-De Vries syndrome (610443) | ***KANSL1*** | 17 | NM_001193465.2: c.1420C>T (p.R474C) |  | PM2, PS2, PP3 | DN | Het |
| 50 | M | 2 | + | + | - | - | Dysmorphic facial features, ventriculomegaly | Paroxysmal nonkinesigenic dyskinesia, 3, with or without generalized epilepsy (609446) | ***KCNMA1*** | 10 | NM_002247.4: c.2984A>G (p.N995S) | CM182525  - | PM2, PP3, PP5 | UD | Het |
| 132 | M | 12 | + | + | + | - | Unique facial features, macrodontia, large protruding ears, sleep disorder | Epileptic encephalopathy (**) | ***KCNC2*** | 12 | NM_139137.4:  c.375C>G (p.C125W) | - | PM2,PS2 | DN | Het |
| 197 | M | 27 | + | + | - | - | Epilptic encephalopathy | Epileptic encephalopathy (**) | ***KCNC2*** | 12 | NM_139137.4:  c.1052G>A (p.R315K) | - | PM2,PP,PS2 | DN | Het |
| 242 | F | 5 | + | + | - | - | Bulbous nasal tip, low anterior hairline, pointed chin, high arched palate, persistent fetal fingertip pads | Developmental and epileptic encephalopathy 14 (614959) | ***KCNT1*** | 9 | ENST00000371757.7: c.1421G>A (p.R474H) | CM129796 | PM2, PM5,PM1, PP5, PS2 | DN | Het |
| 4 | M | 16 | + | + | - | - | Dysmorphic facial features, craniosynostosis | Developmental and epileptic encephalopathy 7 (613720) | ***KCNQ2*** | 20 | NM_172107.4: c.878T>C (p.L293P) | CM184286 | PM1, PP2, PM2, PP3, PS2 | DN | Het |
| 60 | M | 12 | + | + | - | - | Atactic gait | Developmental and epileptic encephalopathy 7 (613720) | ***KCNQ2*** | 20 | NM_172107.4: c.629G>A (p.R210H) | CM1311201 | PM1, PP2, PM2 ,PM5, PP3, PP5, PS2 | DN | Het |
| 18 | M | 15 | + | - | - | - | Dysmorphic facial features, short stature, wide-based gait | *KCNQ3*-related developmental disability (**) | ***KCNQ3*** | 8 | NM_004519.4: c.688C>T (p.R230C) | CM129355 | PM2, PM5, PP3, PP5, PS2 | DN | Het |
| 31 | M | 20 | + | - | + | - | Dysmorphic facial features, microcephaly, macroglossia, ADHD | Wiedemann-Steiner syndrome (605130) | ***KMT2A*** | 11 | NM_005933.4: c.2483C>G (p.S828*) | - | PVS1, PM2, PS2 | DN | Het |
| 174 | F | 9 | + | - | + | + | Atrial septal defect, short stature, dysplaia of vermis, feeding difficulties, right auditory canal atresia and microtia, hypothyroidism, persistant fetal fingertip pads, brachydactyly, sleep disturbance, ADHD | Kabuki syndrome 1 (147920) | ***KMT2D*** | 12 | NM_003482.4: c.7650del (p.V2551Sfs*32) | CD114931 | PVS1, PM2, PS2 | DN | Het |
| 41 | M | 3 | + | - | - | - | Relative macrocephaly | Mental retardation, autosomal dominant 51 (617788) | ***KMT5B*** | 11 | NM_017635.5: c.833A>T (p.N278I) |  | PM2, PP3, PS2 | DN | Het |
| 109 | M | 13m | + | - | - | - | Macrocephaly, broad forehead, hypertelorism, strabismus, short neck | Mental retardation, autosomal dominant 51 (617788) | ***KMT5B*** | 11 | NM_017635.5: c.541C>G (p.H181D) |  | PM2, PP3, PS2 | DN | Het |
| 83 | F | 5 | + | + | + | - | Proptosis, upslanted palpebral fissures, low set ears, FTT, muscle spasticity | Cardiofaciocutaneous (CFC) syndrome 3 (615279) | ***MAP2K1*** | 15 | NM_002755.4: c.124C>T (p.L42F) | CM092997 | PM1, PP2, PM2, PP3, PP5, PS2 | DN | Het |
| 271 | F | 21 | + | + | - | - | Microcephaly, dysmorphic facial features, macrodontia, scoliosis, short palm and fingers, cardiomyopathy, increased nuchal translucency | Cardiofaciocutaneous (CFC) syndrome 3 (615279) | ***MAP2K1*** | 15 | NM_002755.4: c.389A>G (p.Y130C) | CM061104 | PM1, PP2, PM2, PM5, PP3, PP5, PS2 | DN | Het |
| 272 | M | 1.5 | + | - | - | + | FTT, short stature, feeding difficulties, gastroesophageal reflux, distal arthrogryposis, pes valgus, rt. lamboidal plagiocephaly, lt. mild hydronephrosis, lt. lacrimal duct obstruction, hearing impairment, transverse palmar crease, cryptorchidism, polyhydramnios | Impaired intellectual development and distinctive facial features with or without cardiac defects (616789) | ***MED13L*** | 12 | NM_015335.5: c.541_556delinsA (p.V181_H186delinsN) | - | PM2,  PM4, PS2 | DN | Het |
| 269 | M | 2 | + | - | - | + | Atrial septal defect, micropenis | Impaired intellectual development and distinctive facial features with or without cardiac defects (616789) | ***MED13L*** | 12 | NM_015335.5: c.2320del (p.I774Ffs*27) | - | PVS1, PM2, PS2  EX 12/31 | DN | Het |
| 206 | F | 7 | + | + | + | - | Toe clubbing, hyperlaxity, spinal syrinx, strabismus, absent speech, IUGR | Mental retardation, stereotypic movements, epilepsy, and/or cerebral malformations (613443) | ***MEF2C*** | 5 | NM_002397.5:  Deletion of exon 2 (coding exon 1) | - | PM2, PS2 | DN | Het |
| 221 | F | 6 | + | + | - | - | - | Mental retardation, autosomal dominant 39 (616521) | ***MYT1L*** | 2 | ENST00000647738.2: c.1706G>A (p.R569Q) | CM1712150 | PM2, PP2, PP5, PS2 | DN | Het |
| 211 | F | 33 | + | + | - | - | Polycystic kidney disease | Mental retardation, X-linked 98 (300912) | ***NEXMIF*** | X | NM_001008537.3: c.937C>T (p.R313*) | CM174747 | PVS1, PM2, PP5, PS2 | DN | Het |
| 173 | M | 3 | - | - | + | - | Neurofibromas, optic glioma, macrocephaly, scoliosis | Neurofibromatosis, type 1 (162200) | ***NF1*** | 17 | NM_000267.3: c.888+2T>G | CS110504 | PVS1, PM2, PP5, PS2 | DN | Het |
| 280 | M | 2 | + | - | - | - | Dysmorphic facial features, hypotonia, accelerated growth of head circumference | Sotos syndrome (117550) | ***NSD1*** | 5 | NM_022455.5: c.4411C>G (p.R1471G) | CM032972 | PM2, PP3, PP1, PP4 | Inh | Het |
| 20 | M | 2 | + | - | - | - | Ptosis, posterior fossa malformation – Blake’s pouch, plagiocephaly | Mental retardation, X-linked, with cerebellar hypoplasia and distinctive facial appearance (300486) | ***OPHN1*** | X | NM_002547.3: c.170T>A (p.V57D) | - | PM2, PS2 | DN | Hem |
| 180 | M | 30 | + | + | - | - | Dysmorphic facial features, behavioral problems, brain arachnoid cyst | Mental retardation, X-linked, with cerebellar hypoplasia and distinctive facial appearance (300486) | ***OPHN1*** | X | NM_002547.3: c.2159-1G>C | - | PVS1, PM2, PS2 | DN | Hem |
| 118 | M | 6 | + | + | + | - | Bulbous nasal tip, thin upper lip, downslanted palpebral fissures, hypotonia, Mongolian spot, café au lait spots, bilateral pes planus | Schuurs-Hoeijmakers Syndrome (615009) | ***PACS1*** | 11 | NM_018026.4: c.607C>T (p.R203W) | CM1211547 | PM2, PP5,PS2 | DN | Het |
| 127 | M | 1 | + | + | - | - | Lissencephaly, microcephaly | Lissencephaly 1 (607432) | ***PAFAH1B1*** | 17 | NM_000430.4: c.1136A>G (p.H379R) | - | PM2, PM1, PP2, PM5, PP3, PS2 | DN | Het |
| 251 | M | 10 | + | - | - | + | Severe progressive microcephaly, dysmorphic facial features, nasal speech, radio-ulnar synostosis, VSD, right hydronephrosis, right inguinal hernia, cerebellar hypoplasia, post natal growth retardation, short stature, severe IUGR | Congenital anomalies of the kidney and urinary tract syndrome with or without hearing loss, abnormal ears, or developmental delay (617641) | ***PBX1*** | 1 | NM_002585.4: c.320G>C (p.R107P) | - | PM2, PP2, PS2 | DN | Het |
| 47 | F | 2 | + | - | + | - | Microcephaly, severe SGA, transient neonatal hypoglycemia, neonatal teeth, FTT, delayed and abnormal pattern of dentition, hyperlaxity | White Sutton syndrome (616364) | ***POGZ*** | 1 | NM_015100.4: c.3040C>T (p.Q1014*) | CM162601 | PVS1, PM2, PS2 | DN | Het |
| 129 | M | 8 | + | + | + | - | - | Neurodevelopmental disorder and language delay with or without structural brain abnormalities (618354) | ***PPP2CA*** | 5 | NM_002715.4: c.667G>C (p.D223H) | CM190630^4^ | PM2, PM5, PP2, PP5, PS2 | DN | Het |
| 24 | F | 18.5 | + | - | + | - | Macrocephaly, dysmorphic facial features, hypoplasia of vermis on brain MRI | Mental retardation, autosomal dominant 35 (616355) | ***PPP2R5D*** | 6 | NM_180976.3: c.496G>A (p.E166K) | CM153575 | PM1, PP2, PM2, PP5, PS2 | DN | Het |
| 147 | F | 3 | + | + | - | - | Developmental regression, long philtrum, white matter changes on brain MRI | Mental retardation, autosomal dominant 35 (616355) | ***PPP2R5D*** | 6 | NM_180976.3: c.619T>A (p.W175R) | CM147778 | PS1, PM1, PP2, PM2, PP3, PP5, PS2 | DN | Het |
| 78 | F | 31 | + | - | - | + | Dysmorphic facial features, velopharyngeal insufficiency, broad neck, pulmonic stenosis, short stature, splenomegaly, coagulation disorder | Noonan symdrome 1 (163950) | ***PTPN11*** | 12 | NM_001330437.2: c.923A>G (p.N308S) | CM021135 | PS1, PM5, PM2, PM1, PP2, PP5, PS2 | DN | Het |
| 61 | M | 2 | + | - | - | + | Bilateral retinoblastoma, postaxial polydactyly, cryptorchidism | Retinoblastoma (180200) | ***RB1*** | 13 | NM_000321.3: c.2359C>T (p.R787*) | CM900196^5^ | PVS1, PM2, PP5, PS2 | DN | Het |
| 265 | M | 2 | + | - | - | - | Dysmorphic facial features, microcephaly, strabismus, hearing impairment, hypotonia, pectus carinatum | Coffin-Lowry syndrome (303600) | ***RPS6KA3*** | X | NM_004586.3: c.1152delG (p.F385Lfs*40) | - | PVS1, PM2, PP4 | Inh | Hem |
| 76 | F | 25 | + | + | + | - | Strabismus, marfanoid habitus, clubfoot, OCD | Glass syndrome (612313) | ***SATB2*** | 2 | NM_015265.4: c.1285C>T (p.R429*) | CM1711207 | PVS1, PM2, PP5, PS2 | DN | Het |
| 14 | F | 37 | + | + | - | - | Dysmorphic facial features | Dravet syndrome (607208) | ***SCN1A*** | 2 | ENST00000674923.1: c.5300T>A, (p.V1767D) | - | PM1, PP2, PM2, PP3, PS2 | DN | Het |
| 227 | M | 2 | + | - | - | - | Fleshy upturned lobules | Developmental and epileptic encephalopathy 11 (613721) | ***SCN2A*** | 2 | NM_021007.3: c.2562+2T>C | - | PM2, PP5, PS2 | DN | Het |
| 34 | M | 3 | + | - | - | - | Dysmorphic facial features, strabismus, abnormal brain MRI findings | Developmental and epileptic encephalopathy 13 (614558) | ***SCN8A*** | 12 | NM_014191.4: c.4064delA, (p.Y1355Sfs*38) | - | PVS1, PM2, PS2 | DN | Het |
| 232 | M | 8 | - | - | - | + | Dysmorphic facial features, pear-shaped nose, protruding ears, dental anomalies, congenital nystagmus, skeletal findings, pectus excavatum, short stature, hydronephrosis | Short stature, facial dysmorphism, and skeletal anomalies with or without cardiac anomalies (619184) | ***SCUBE3*** | 6 | NM_152753.4: c.2785C>T, (p.R929*) | ^6^ | PM2, PVS1, PP1, PP4, PP5 | Inh | Hom |
| 15 | F | 4 | + | + | - | - | Dysmorphic facial features, hydronephrosis | Mental retardation, autosomal dominant 29 (616078) | ***SETBP1*** | 18 | NM_015559.3: c.1777C>T (p.Q593*) | - | PVS1, PM2, PS2 | DN | Het |
| 126 | F | 6 | - | - | - | - | Cerebellar ataxia, frontal bossing, sparse hair, verbal dyspraxia, small wide spaced teeth, white matter changes on brain MRI | Epilepsy, early-onset, with or without developmental delay (618832) | ***SETD1A*** | 16 | NM_014712.3: c.4582-2_4582-1del | - | PM2, PS2 | DN | Het |
| 259 | F | 24 | + | + | - | - | ADHD, short stature, hypothyroidism | Luscan-Lumish syndrome (616831) | ***SETD2*** | 3 | ENST00000409792.4: c.1748_1751del (p.K583Sfs*17) | - | PVS1, PM2, PS2 | DN | Het |
| 95 | M | 5 | + | - | + | - | Microcephaly, long philtrum with thin upper lip, ADHD | Phelan-McDermid syndrome (606232) | ***SHANK3*** | 22 | NM_001372044.2: c.4608G>A (p.W1536*) | - | PVS1, PM2, PS2 | DN | Het |
| 193 | M | 7 | + | + | - | - | Relative microcephaly, spasticity, brain atrophy on MRI | Spastic tetraplegia, thin corpus callosum, and progressive microcephaly (616657) | ***SLC1A4*** | 2 | NM_003038.5: c.766G>A (p.E256K) | CM155873 | PM2, PP5 | Inh | Hom |
| 72 | M | 32 | + | - | - | - | Dysmorphic facial features, short stature, tetralogy of Fallot, diabetes mellitus, ADHD | Myhre syndrome (139210) | ***SMAD4*** | 18 | ENST00000342988.8: c.1498A>G (p.I500V) | CM1110610 | PM1, PP2, PM2, PM5, PP3, PP5, PS2 | DN | Het |
| 53 | F | 3 | + | - | - | - | Microcephaly, dysmorphic facial features | Cornelia de Lange Syndrome 2 (300590) | ***SMC1A*** | X | NM_006306.4: c.2341T>C (p.C781R) | - | PM2, PM1, PP2, PM5, PP3, PS2 | DN | Het |
| 103 | F | 17 | + | + | - | - | Cataplexy, peripheral demyelinating neuropathy, ataxia, kyphosis, dyscalculia | Cornelia de Lange Syndrome 2 (300590) | ***SMC1A*** | X | NM_006306.4: c.1171C>T (p.Q391*) | - | PVS1, PM2, PS2 | DN | Het |
| 111 | F | 15 | + | + | - | - | Microcephaly, hirsutism, fifth finger brachydactyly, behavior problems, white matter changes on brain MRI, ADHD | Cornelia de Lange Syndrome 2 (300590) | ***SMC1A*** | X | NM_006306.4: c.802_804del (p.K268del) | CD096588 | PM2, PP5 , PS2 | DN | Het |
| 57 | M | 1 | - | - | - | + | Relative large head circumference, cleft palate, hearing impairment, prominent brachydactyly, umbilical hernia, short stature | Acamplomelic campomelic dysplasia (114290) | ***SOX9*** | 17 | NM_000346.4: c.529C>T (p.R177W) | CM210126 | PM2, PM1, PP2, PP3, PP1 | Inh | Het |
| 253 | M | 12 | + | - | + | - | Microcephaly, dysmorphic facial features, sunken eyes, protruding ears, persistent fetal fingertip pads, ADHD | Congenital disorder of glycosylation, type Iy  (300934) | ***SSR4*** | X | NM_001204526.1: c.294+2_294+8del | - | PVS1, PM2, PS2 | DN | Hem |
| 94 | M | 3 | + | + | - | - | Smooth philtrum, anteverted nostrils, pilonidal dimple, perianal abcess | Developmental and epileptic enceophalopathy 4 (612164) | ***STXBP1*** | 9 | NM_001374306.2: c.569+1G>A | CS208256 | PM2, PP5, PS2 | DN | Het |
| 162 | M | 1 | + | + | - | - | Hypotonia, hearing impairment | Developmental and epileptic enceophalopathy 4 (612164) | ***STXBP1*** | 9 | NM_001374306.2: c.236C>T (p.P79L) | CM160285 | PM2, PP3, PP5, PS2 | DN | Het |
| 33 | F | 5 | + | + | - | - | Broad forehead | Mental retardation, autosomal dominant 5 (612621) | ***SYNGAP1*** | 6 | NM_006772.3: c.1167_1168delAG  (p.G391Qfs*27) | CD190428 | PVS1, PM2, PP5, PS2 | DN | Het |
| 82 | M | 6 | + | + | - | - | ADHD | Baker-Gordon syndrome (618218) | ***SYT1*** | 12 | NM_005639.3: c.1198C>T (p.R400*) | - | PM2, PS2 | DN | Het |
| 112 | M | 11 | + | + | - | - | Thick eyebrows, large protruding ears, bilateral fifth finger clinodactyly, ADHD, behavior problems | Mental retardation, autosomal dominat 41 (616994) | ***TBL1XR1*** | 3 | NM_024665.7: c.597_600: delTGAG (p.S199Rfs*10) | - | PVS1, PM2,PS2 | DN | Het |
| 19 | M | 49 | + | + | - | - | Relative macrocephaly | Intellectual developmental disorder with autism and speech delay (606053) | ***TBR1*** | 2 | NM_006593.4: c.1132A>T (p.T378S) | - | PM2, PP3, PS2 | DN | Het |
| 5 | M | 12 | + | + | - | - | Dysmorphic facial features, ventriculomegaly | Pitt-hopkins syndrome (610954) | ***TCF4*** | 18 | NM_003199.3: c.1805C>T (p.T602I) | - | PM2, PM1, PP3, PS2 | DN | Het |
| 148 | F | 6 | + | - | + | - | Microcephaly, absent speech, hypotonia, deep set eyes, cupped ears, widely spaced teeth, bruxism, persistent fetal fingertip pads, stereotypic hand movements | Pitt-hopkins syndrome (610954) | ***TCF4*** | 18 | NM_003199.3: c.1146+1G>A | CS091867 | PVS1, PM2, PP5, PS2 | DN | Het |
| 39 | F | 10 | + | + | + | - | Dysmorphic facial features, motor dyspraxia | Developmental delay with variable intellectual impairment and behavioral abnormalities (618430) | ***TCF20*** | 22 | NM_005650.4: c.5221_5222del (p.R1741Gfs*9) | - | PVS1, PM2, PS2 | DN | Het |
| 182 | F | 26 | + | + | - | - | Arthropathy | Developmental delay with variable intellectual impairment and behavioral abnormalities (618430) | ***TCF20*** | 22 | NM_005650.4: c.3849_3850insTC (p.L1284Sfs*68) | - | PVS1, PM2, PS2 | DN | Het |
| 89 | F | 2 | + | - | - | - | Strabismus, eyelid puffiness | Mental retardation, autosomal dominant 49 (617752) | ***TRIP12*** | 2 | NM_001348330.2: c.2378_2379insT (p.V794Sfs*2) | - | PVS1, PM2, PS2 | DN | Het |
| 186 | M | 3 | + | - | - | - | Hypotonia, suspected hearing impairment, strabismus, thrombocytosis, fifth finger clinodactyly, joint hypermobility, breath holding spells, persistent fetal fingertip pads | Mental retardation, autosomal recessive 68 (618302) | ***TRMT1*** | 19 | ENST00000357720.9: c.1332_1333del (p.Y445Lfs*28)  / c.232C>T, (p.Q78*) | CD159154  - | PVS1, PM2, PP5  PVS1, PM2, PM3 | Inh  Inh | Comp Het |
| 81 | F | 12 | + | - | - | - | Microcephaly, dysmorphic facial features, beaked nose, narrow nasal bridge, mild bilateral renal hypoplasia, new onset diabetes, fifth finger clinodactyly, ADHD | Microcephaly, short stature, and impaired glucose metabolism 1, (616033) | ***TRMT10A*** | 4 | NM_001375882.1: c.616G>A (p.G206R) | CM148063^7^ | PM2, PP3, PP5 | Inh | Hom |
| 62 | M | 5.5 | + | - | - | - | Microcephaly, dysmorphic facial features | Pontocerebellar hypoplasia type 2A (277470) | ***TSEN54*** | 17 | NM_207346.3: c.371G>T (p.G124V) | CM110074 | PM2, PP3, PP5 | Inh | Hom |
| 194 | M | 18 | + | + | + | - | Microcephaly, short stature, white matter abnormalities in brain MRI, ADHD | Mental retardation, X-linked 58 (300210) | ***TSPAN7*** | X | NM_004615.4: c.289del (p.L97Wfs*8) | - | PVS1, PM2 | Inh | Hem |
| 161 | M | 3.5 | + | - | - | - | CP-like disorder, hip dysplasia, spastic paraplegia | Lissencephaly 3 (611603) | ***TUBA1A*** | 12 | NM_006009.4:c.431G>T (p.G144V) | - | PM1, PP2, PM2, PP3, PS2 | DN | Het |
| 70 | F | 28 | - | - | - | + | Craniosynostosis, microcephaly, ptosis, choanal atresia, bliateral hearing impairment, hypotonia, GERD, hypertrichosis, normal cognition | Saethre-Chotzen syndrome with or without eyelid anomalies (101400) | ***TWIST1*** | 7 | NM_000474.4: c.437_448del, (p.I146_L149del) | - | PM2, PM4, PM1, PS2 | DN | Het |
| 141 | M | 1.5 | + | - | - | - | Microcephaly, strabismus | Angelman syndrome (105830) | ***UBE3A*** | 15 | NM_130839.5: c.1682G>A (p.G561E) | CM1912264 | PM2, PP3, PS2 | DN | Het |
| 177 | M | 6m | - | - | - | + | Hypospadias, microrethrognathia, recurrent apneas, sinus vein thrombosis, mild hypercalcemia, mildly thin and dysplastic CC, IUGR | Hao-Fountain syndrome (616863) | ***USP7*** | 16 | NM_003470.3: c.1175G>C (p.G392A) | - | PM2, PS2 | DN | Het |
| 131 | F | 1 | + | - | - | + | Microcephaly, right posterior plagiocephaly, right single transverse palmar crease, congenital metatarsus varus, atrial septal defect secundum, hypoplasia of labia minora | Cohen syndrome (216550) | ***VPS13B*** | 8 | NM_017890.5: c.3984G>A (p.W1328*) /  c.11906_11916delinsG (p.P3969Rfs*41) | -  - | PM2, PP5, PM3  PVS1, PM2, PM3 | Inh | Comp Het |
| 254 | F | 1.5 | + | + | - | - | Early closure of fontanels | Neurodegeneration with brain iron accumulation, 5 (300894) | ***WDR45*** | X | ENST00000376372.9: c.827+1G>A | CS1211569 | PM2, PP5, PS2 | DN | Het |
| 91 | M | 8.5 | + | + | - | - | Upslanted palpebral fissures, axial hypotonia, ptosis, left-kidney hydronephrosis, ADHD | Developmental and epileptic encephalopathy 56 (617665) | ***YWHAG*** | 7 | NM_012479.4: c.169C>T (p.R57C) | CM203972 | PM2, PP3, PS2 | DN | Het |
| 185 | F | 5 | + | + | + | - | Microcephaly, short broad first toes, short first fingers, left hand single transverse palmar crease, paraplegia | Wieacker-Wolff Syndrome, Female-restricted (301041) | ***ZC4H2*** | X | ENST00000374839.8: c.575G>A (p.C192Y) | - | PM2, PM1, PP2, PS2 | DN | Het |
| 208 | F | 50 | + | + | - | - | Dysmorphic facial features | Mowat-Wilson syndrome (235730) | ***ZEB2*** | 2 | NM_014795.4: c.3242G>A (p.C1081Y) | - | PM2, PM5, PM1, PS2 | DN | Het |
| 104 | M | 1.5 | + | - | - | - | Laryngomalacia, FTT, bruxism, persistent fetal fingertip pads | Neurodevelopmental disorder with movement abnormalities, abnormal gait, and autistic features (617865) | ***ZSWIM6*** | 5 | NM_020928.2: c.2737C>T (p.R913*) | CM1716634 | PVS1, PM2, PP,5 PS2 | DN | Het |

Variant were classified according to ACMG criteria by the authors, using Varsome (<https://varsome.com>) and Franklin by genoox (<https://franklin.genoox.com/clinical-db/home>). Some criteria were edited or added manually. When the same specific variant was reported in HGMD as disease-causing, it was considered as additional strong or moderate criteria.

ACMG, American College of Medical Genetics and Genomics; ADHD, attention deficit-hyperactivity disorder; ASD, autism spectrum disorder; AVSD, atrio-ventricular septal defect; CC, corpus callosum; Comp Het, compound heterozygous; CK, creatinie kinase, CP, cerebral palsy; DD, developmental delay; DN, de-novo; ES, exome sequencing; F, female; FTT, failure to thrive; GDD, global developmental delay; GERD, gastro-esophageal reflux disease; Hem, hemizygous; Het, heterozygous; HGMD, human gene mutation database; Hom, homozygous; ID, intellectual disability; IUGR, intrauterine growth retardation; M, male; MCA, multiple congenital anomalies; Mo, month; MRI, magnetic resonance imaging; OCD, obsessive-compulsive disorder; pLI, probability of being loss-of-function intolerant; SGA, small for gestational age; UD, undetermined, VSD, ventriculoseptal defect

^1^ Patient reported in: Pode-Shakked N, et al. BRPF1-associated intellectual disability, ptosis, and facial dysmorphism in a multiplex family. Mol Genet Genomic Med 2019;7(6):e665.

^2^ Patient reported in: Rodan LH, et al. Phenotypic expansion of *CACNA1C*-associated disorders to include isolated neurological manifestations. Genet Med 2021 [Epub ahead of print].

^3^ Patient reported in: Rodríguez-Palmero A, et al. DLG4-related synaphtopathy: A new rare brain disorder. Genet Med 2021;23(5):888-899.

^4^ Patient reported in: Reynhout S, et al. De novo mutations affecting the catalytic Cα subunit of PPSA, PPP2CA, cause syndromic intellectual disability resembling other PP2A-related neurodevelopmental disorders. Am J Hum Genet 2019;104(1):139-156.

^5^ Phenotype only partially explained by *RB1* variant.

^6^ Patient reported in: Lin YC, et al. SCUBE3 loss-of-function causes a recognizable recessive developmental disorder due to defective bone morphogenetic protein signaling. Am J Hum Genet 2021;108(1):115-133.

^7^ Patient reported in: Stern E, et al. *TRMT10A* mutation in a child with diabetes, short stature, microcephaly and hypoplastic kidneys. J Clin Res Pediatr Endocrinol 2021 [Epub ahead of print].

** Phenotype not yet annotated in OMIM.
